# OPUSeq simplifies detection of low-frequency DNA variants and uncovers fragmentase-associated artifacts

**DOI:** 10.1093/nargab/lqac048

**Published:** 2022-06-27

**Authors:** Alisa Alekseenko, Jingwen Wang, Donal Barrett, Vicent Pelechano

**Affiliations:** SciLifeLab, Department of Microbiology, Tumor and Cell Biology, Karolinska Institutet, Tomtebodavägen 23A, 17165, Solna, Sweden; SciLifeLab, Department of Microbiology, Tumor and Cell Biology, Karolinska Institutet, Tomtebodavägen 23A, 17165, Solna, Sweden; SciLifeLab, Department of Microbiology, Tumor and Cell Biology, Karolinska Institutet, Tomtebodavägen 23A, 17165, Solna, Sweden; SciLifeLab, Department of Microbiology, Tumor and Cell Biology, Karolinska Institutet, Tomtebodavägen 23A, 17165, Solna, Sweden

## Abstract

Detection of low-frequency DNA variants (below 1%) is becoming increasingly important in biomedical research and clinical practice, but is challenging to do with standard sequencing approaches due to high error rates. The use of double-stranded unique molecular identifiers (dsUMIs) allows correction of errors by comparing reads arising from the same original DNA duplex. However, the implementation of such approaches is still challenging. Here, we present a novel method, one-pot dsUMI sequencing (OPUSeq), which allows incorporation of dsUMIs in the same reaction as the library PCR. This obviates the need for adapter pre-synthesis or additional enzymatic steps. OPUSeq can be incorporated into standard DNA library preparation approaches and coupled with hybridization target capture. We demonstrate successful error correction and detection of variants down to allele frequency of 0.01%. Using OPUSeq, we also show that the use of enzymatic fragmentation can lead to the appearance of spurious double-stranded variants, interfering with detection of variant fractions below 0.1%.

## INTRODUCTION

Biomedical research and personalized medicine increasingly rely on our ability to quickly and precisely determine DNA sequences and their associated mutations or polymorphisms. Standard next-generation sequencing allows us to accurately determine variants above 1% variant allele fraction (VAF) (1). However, the ability to confidently detect variants present at far lower fractions is important for multiple areas of research, such as studies of pathogen intrahost variation (2,3), mitochondrial heteroplasmy (4–6) or mechanisms of mutagenesis (7,8). Detection of low-frequency variants is also important in the clinical setting, aiding in cancer patient stratification and treatment selection. Intratumoral heterogeneity is widely recognized as an important factor influencing patient outcomes (9,10), one of the reasons being that heterogeneous tumors can harbor drug-resistant cells. Such cells may be present at a very low fraction in a tumor and thus elude detection. After successful cancer treatment, the detection of minimal residual disease (MRD) is of high clinical relevance, as identification of the scarce remaining cancer cells in blood can be vital for predicting relapse (11,12).

Many methods have been developed to improve our ability to accurately detect low-frequency variants (1). In order to do so, errors arising during sequencing and library preparation, as well as DNA damage due to sample handling, need to be eliminated from the data. If the sequencing is performed at high depth, one can compare several reads arising from the same original DNA fragment and retain only variants found in the majority of the reads. Adding unique molecular identifiers (UMIs)—short random sequences—to the ends of DNA introduces barcodes that help to trace each read back to the original fragment (13,14). Single-stranded UMIs are easy to incorporate into a library, but can only group reads arising from the same strand and thus do not allow elimination of errors arising from early cycles of PCR or from DNA damage (affecting all reads from one of the strands). In 2012, the duplex sequencing (Duplex-seq) approach (15) introduced double-stranded UMIs (dsUMIs), which enabled pairing of the two strands of the same original fragment, thus theoretically reducing the error rate to <10^–9^ (1). However, Duplex-seq involves complicated and failure-prone synthesis of adapters carrying the dsUMIs (16). Several alternative approaches have been published in recent years (17–22). Some have replaced random UMIs with simpler, predefined barcodes, which are easier to incorporate into library adapters but might limit the ability to accurately identify extremely low variant fractions (19,20,22). SaferSeqS (21) improved on the process of dsUMI addition, removing the need to pre-synthesize adapters. However, the library preparation strategy is not straightforward and contains multiple additional steps and enzymes compared to standard protocols.

Thus, there remains a need for simpler approaches to accurately detect very low variant fractions while correcting for strand-specific sequencing errors originating from the library preparation or sample handling steps. Here, we introduce a novel way to incorporate dsUMIs into DNA sequencing libraries using simple, commercially synthesized adapters and a two-stage PCR with a standard polymerase. This method, which we call one-pot dsUMI sequencing (OPUSeq), creates dsUMI-labeled libraries from a range of genomic DNA (gDNA) inputs and is compatible with standard PCR-based library preparation and target enrichment approaches. We demonstrate the ability of OPUSeq to efficiently remove errors and detect low-frequency variants down to 0.01% VAF. We also report a new type of fragmentase-induced artifacts, which could only be uncovered using a duplex error correction strategy. These artifacts appear at VAF of up to 0.14% and may thus interfere with low-frequency variant calling.

## MATERIALS AND METHODS

### Human gDNA

Purified gDNA from the HapMap project genotypes was obtained from the Coriell Institute (NA12751 and NA12814). For samples captured on the 56-kb cancer gene panel, we used 5 ng NA12814 DNA. For the VAF series experiments, we used either 1 μg (with the fragmentase approach) or 200 ng (with the sonication approach) mixed-genotype DNA. We mixed DNA at 1%, 0.1%, 0.05%, 0.01% and 0% VAF of ‘test’ genotype (NA12751) in ‘background’ genotype (NA12814). Since the single-nucleotide polymorphisms (SNPs) of interest were heterozygous in NA12751, fractions by mass were two times higher than those by allele. We prepared mixes in duplicate and diluted in IDTE buffer (10 mM Tris–HCl, pH 8, 0.1 mM EDTA). We split each replicate to prepare the library according to either OPUSeq protocol or standard KAPA protocol (KAPA HyperPlus or HyperPrep Kits, Roche). To obtain neonatal fibroblast DNA, we extracted DNA from cultured Hs27 cells (ATCC CRL-1634) of passage 2 using the Quick-DNA MiniPrep Plus Kit (Zymo Research) according to manufacturer’s instructions. We performed all measurements of DNA concentration using the Qubit dsDNA HS Assay Kit (Thermo Fisher).

### Sonication

For the KAPA HyperPrep Kit, gDNA was sonicated to 150–200 bp fragments using a Covaris ME220 instrument. For the experiment comparing fragmentation methods and DNA sources, DNA was sonicated in microTUBE AFA Fiber Pre-Slit Snap-Cap 6 × 16 mm tubes at 10 ng/μl in TE buffer (10 mM Tris–HCl, 1 mM EDTA) for 225 s at peak power of 75 W, 25% duty factor and 1000 cycles per burst. For the VAF series experiment, DNA was sonicated prior to mixing the two genotypes, at 50 ng/μl in TE buffer in 8 microTUBE AFA Beads Strip V2 for 140 s at peak power of 50, 30% duty factor and 50 cycles per burst. After sonication, we purified DNA with 1.8× volume ratio of AMPure XP beads (Beckman Coulter) and eluted into IDTE.

### Library preparation using the standard KAPA protocols

We used KAPA HyperPlus and HyperPrep Kits according to manufacturer’s instructions with minimal variations. During the HyperPlus protocol, we performed fragmentation for 20 min at 37°C and then inactivated fragmentase at 70°C for 15 min. We ligated QIAGEN QIAseq FX 24-plex dual-indexed adapters to DNA for 1 h at 20°C. For 1 μg input, we added 10 μl of 15 μM adapter instead of the usual 5 μl to maximize ligation efficiency. We performed 2 cycles of PCR for 1 μg input and 10 cycles for 5 ng input. The final concentration of Library Amplification Primer Mix was 1 μM for 5 ng DNA input and 2 μM for 1 μg DNA input.

For the HyperPrep protocol, we used 200 ng of Covaris-sonicated DNA in IDTE (see earlier) as input. We ligated either QIAGEN QIAseq FX 24-plex dual-indexed adapters or KAPA dual-indexed adapters (Roche) to DNA for 1 h at 20°C. Finally, we performed PCR for four or five cycles with 1 μM Library Amplification Primer Mix.

For the test of different fragmentation and end-repair (ER) protocols, we used two different ER enzyme mixes. These two mixes were both included with the KAPA HyperPlus Kit. One of them (ER mix 1, tube with purple cap) is the mix that the HyperPlus Kit contained originally and is the same as in the HyperPrep Kit. The other mix (ER mix 2, orange cap) is a newer addition, claimed to be optimized for the fragmentase-based HyperPlus protocol. For the VAF dilution experiment using HyperPlus, we used ER mix 1.

### OPUSeq

**Figure 1.**
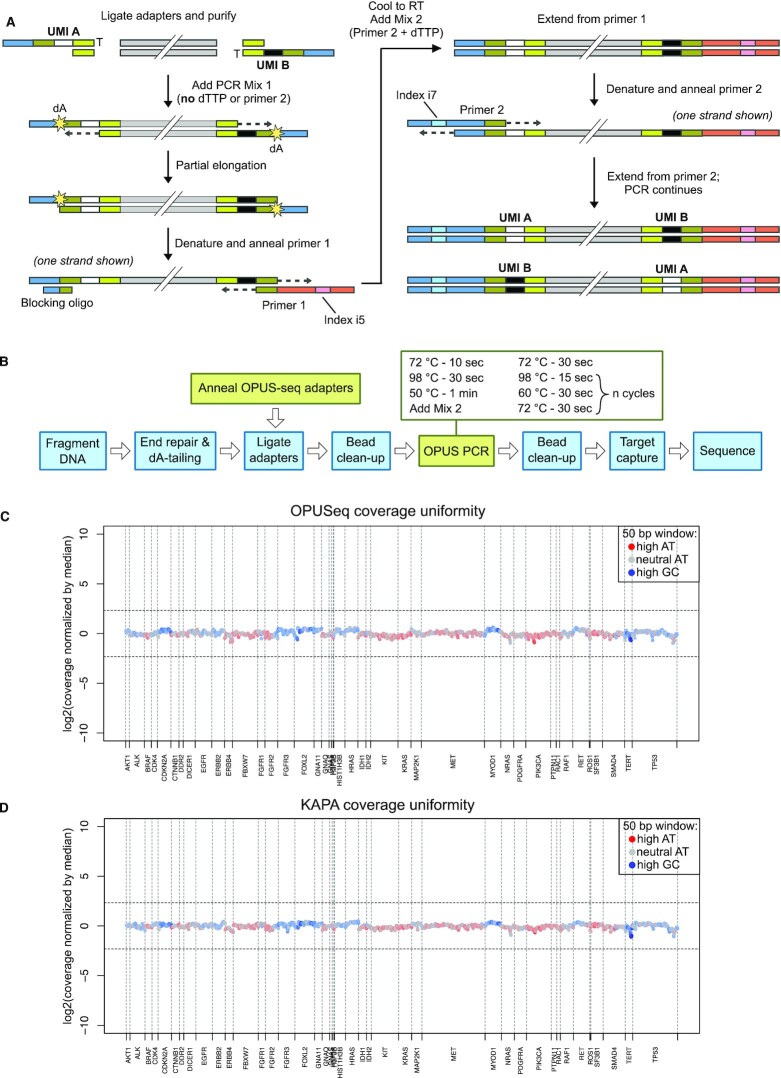
OPUSeq workflow and performance in target capture. (**A**) Fragmented, end-repaired and dA-tailed DNA is ligated to OPUSeq adapters. Each ligated adapter carries a different single-stranded UMI. The ligated DNA is mixed with PCR mix 1 and subjected to partial elongation, which copies the UMI over to the opposite strand. The elongation stops at the first dA due to lack of dTTP in mix 1. Next, DNA is denatured and primer 1 and blocking oligo are annealed. The reaction is brought to room temperature and primer 2 and dTTP are added (mix 2). Another elongation step ensures full extension of primer 1. Finally, the desired number of standard PCR cycles are performed. (**B**) Schematic showing how OPUSeq is integrated into a standard PCR-based library preparation approach (KAPA). (**C**) Coverage uniformity for an OPUSeq library made from 5 ng gDNA and captured on a 56-kb cancer-related gene panel with a 22-kb target region. Coverage was calculated in bins of 50 bp, normalized by cross-panel median, log_2_ transformed and plotted per bin. Color indicates GC content of each bin. *X*-axis is labeled with the names of the targeted genes. (**D**) Coverage uniformity as in panel (C), but for a library generated from 5 ng gDNA following the standard KAPA HyperPlus protocol.

See Figure 1 for an overview of the OPUSeq workflow. The oligonucleotides (oligo) required for OPUSeq are listed in Supplementary Table S1 and can be obtained from any commercial manufacturer (we used Sigma-Aldrich and Integrated DNA Technologies). In addition to custom adapters, custom PCR primer 1, sequencing read 1 primer and a blocking oligo are required. In order to ensure no dA occurrences in the adapter before the desired elongation endpoint, we omitted dA bases in the UMI and introduced two base substitutions to the standard Illumina sequences. On the other hand, PCR primer 2 has the standard Illumina sequence. To prepare OPUSeq adapters, we annealed Adapter_Fw_[1–4] and Adapter_Rv_[1–4] oligos (each at 48 μM) pairwise (Fw_1 + Rv_1, etc.) in annealing buffer (40 mM Tris–HCl, pH 8, 50 mM NaCl) by heating up to 95°C for 5 min and then cooling down slowly to 10°C. We then mixed the four annealed adapters together in equimolar amounts. Annealed adapters were kept at −20°C and thawed on ice.

OPUSeq libraries were prepared using the KAPA HyperPlus or HyperPrep Kit, with the following changes to the standard protocol. We used OPUSeq annealed adapter mix for ligation (10 μl of 48 μM mix for 1 μg DNA, 5 μl of 15 μM mix for other input amounts) at 20°C for 1 h. After the standard post-ligation bead cleanup, we performed a second cleanup with a 1.8× ratio of AMPure XP beads. This additional cleanup was important for obtaining good library conversion with OPUSeq. We speculate this might be due to a decreased carryover of contaminants from prior reactions. We performed PCR using KAPA HiFi, which possesses strand-displacing activity and is *not* heat-activated (non-hot-start), both of which are requirements for a polymerase compatible with OPUSeq PCR.

When using 5 or 200 ng DNA input, we mixed 20 μl purified DNA with 10 μl KAPA HiFi Fidelity Buffer (5×) (Roche), 2.5 μl blocking oligo (20 μM), 0.75 μl three-dNTP mix (dATP, dGTP and dCTP at 20 mM each), 2.5 μl PE1_MM_MPX_[index#] primer (PCR primer 1, 20 μM), 1 μl KAPA HiFi enzyme (Roche) and 10 μl nuclease-free water. We ran this mix through the pre-incubation consisting of 72°C for 10 s, 98°C for 30 s and 50°C for 1 min, and then returned it to room temperature. We added 0.75 μl dTTP (20 mM) and 2.5 μl PE2_MPX_[index#] primer (PCR primer 2, 20 μM) to the reaction. With 1 μg DNA input, we used 2-fold higher stock concentrations of the blocking oligo and primers. Further, we incubated the reaction at 72°C for 30 s, and ran though *N* cycles of 98°C for 15 s, 60°C for 30 s and 72°C for 30 s. *N* was 11 cycles for 5 ng DNA input, 4–5 cycles for 200 ng DNA input and 4 cycles for 1 μg DNA input. After cycling, the last step was an extension at 72°C for 1 min. Finally, we purified PCR products with 1× AMPure XP beads.

### Hybridization target capture

To optimize our protocol and test capture efficiency, we performed hybridization target capture using a probe panel spanning 56 kb, which covered a set of clinically relevant genes implicated in cancer. For the VAF series experiments, we designed a probe set covering a small part of the HRAS gene (427 bases in length). The set consists of nine 5′ biotinylated 85–86-nt oligo probes. Five of the oligos cover the target region end-to-end with no gaps, while the other four oligos overlap two of the primary five in a staggered design (see figures and Supplementary Table S1). We mixed and diluted the oligo probes in water to achieve a total concentration of 0.75 μM (0.083 μM of each oligo).

We performed hybridization target capture using the xGen Lockdown Reagents from Integrated DNA Technologies according to the manufacturer’s protocol with minimal variations. We varied the amount of whole-genome library used for capture depending on the original input DNA amount. Where possible, we pooled libraries from the same protocol in batches to decrease sample handling. When performing double capture with the HRAS probe set, we hybridized probes overnight (16 h) both times. After the first round of capture and subsequent PCR, we pooled all libraries from the same experiment and performed a second round of capture following the same protocol.

### Sequencing

We sequenced pooled captured libraries paired-end on NextSeq 500 (Illumina) with 151 cycles per read. A custom read 1 sequencing primer (R1_seq_MM in Supplementary Table S1) with one substitution is required to sequence OPUSeq libraries. We spiked in this primer into the standard read 1 primer position on the NextSeq 500/550 v2.5 cartridge at 0.3 μM. This setup allows for simultaneous sequencing of OPUSeq and standard Illumina libraries in the same flow cell. Spiking in custom primers is possible on all Illumina instruments except iSeq100, where it is still possible to use a self-prepared mix containing both standard and custom read 1 primers. This does not affect the quality or output of sequencing.

### Data analysis

For OPUSeq data analysis, we extracted the first six bases of each read as the UMI and added them to the read name using UMI-tools (v. 1.0.0). We then trimmed the reads to remove forward and reverse adapter sequences using cutadapt (v. 3.1). Trimmed reads were mapped to the human genome (hg19) using BWA aln (v. 0.7.17). Further, we corrected the UMIs in mapped reads using the default grouping algorithm of UMI-tools with edit distance set to 3. We wrote a custom script in python (v. 3.7.2) (correct_pair.py) to replace the UMIs in read names with the UMI-tools-corrected UMIs and to filter for convergently mapped read pairs (FR or RF) and mapping quality (MAPQ ≥ 36).

Next, we ran the custom python script consensus_read.py to form consensus sequences. We based this script on UnifiedConsensusMaker.py from the Duplex-seq project (https://github.com/Kennedy-Lab-UW/Duplex-Seq-Pipeline), but modified it for our purpose. In Duplex-seq, FASTQ reads are first grouped based on UMIs, consensus is formed and then consensus sequences are mapped. Since we wanted to also use the information of genomic boundaries, we formed consensus from mapped reads. We grouped reads into tag families based on (i) genomic boundary (start and end coordinates); (ii) final UMI, composed of the two six-base UMIs from each end of the fragment; and (iii) orientation and read number. Each original DNA duplex molecule can thus give rise to four tag families (Supplementary Figure S1A). For consensus making, we kept only families with three or more reads. Although including families with one or two reads can be advantageous in some circumstances (23), we have found that in our data this did not substantially increase duplex recovery. Sometimes, reads belonging to the same family can have different lengths. We therefore added ‘*N*’ to all reads shorter than the longest read until they reached this maximum length (Supplementary Figure S1B). To form the single-stranded consensus sequences (SSCS), we counted base calls from each read in a family at each position. If the same base was called in over two-thirds of reads, this base was written to the consensus. If no base call reached the threshold, *N* was written. Further, to form duplex consensus sequences (DCS), we compared SSCS from opposite strands (see later and Supplementary Figure S1A). A base was kept in DCS only if it was found in both compared SSCS (otherwise, the call was *N*). The final SSCS and DCS were written out in FASTQ format. DCS recovery was calculated as follows: number of DCS/(number of SSCS/2).

Next, we mapped SSCS and DCS FASTQ files to hg19 genome using bowtie2 (v. 2.3.5.1) in local mode with no penalty or ceiling on ambiguous characters (*N*) in reads. In the VAF series dataset with sonication, we used BamUtil trimBam (v. 1.0.15) to trim seven bases from ends of mapped reads. From all the mapped reads, we generated pileup files using samtools mpileup (v. 1.14). When creating pileups of initial filtered mapped files, we set a threshold of 30 on Phred quality score. Finally, we used the script collapse_pileup.py to count reference and variant bases in mapped filtered, SSCS and DCS BAM files. We only considered substitutions (not insertions or deletions). Reads from standard KAPA libraries were directly mapped to the human genome (hg19) using BWA aln and processed with the correct_pair.py script. Pileups and base count files were created in the same way as for OPUSeq filtered mapped reads.

We processed the base count tables resulting from collapse_pileup.py in R (v. 4.0.0). We focused only on the target region from chr11:534034 to chr11:534465. The germline variant in genotype NA12814 (chr11:534332 G>A) was excluded from all analysis and plots. Variant incidence was calculated after excluding the spiked-in NA12751 variants as follows: number of variant bases/total number of bases, at each analysis level (filtered, SSCS and DCS). *P*-values were calculated by comparing each defined VAF sample to the sample containing pure NA12814 genotype. We counted reference and variant bases at each position where there was a variant with a higher frequency in the ‘test’ sample than the ‘background’ sample. We performed the chi-squared test and then Benjamini and Hochberg adjustment on the *P*-values. We set the significance threshold at false discovery rate ≤0.05. To assess capture uniformity, we divided the 56-kb panel target region (22 kb) into 50-bp bins and computed raw coverage by counting the number of reads mapping in each bin. The coverage was then normalized by dividing by median coverage across target region, and log_2_ values were plotted for each bin.

## RESULTS

### Development of OPUSeq

To complement current approaches for detection of low-frequency variants, we developed a simplified method that simultaneously queries both strands of each DNA molecule. We aimed to integrate the addition of a dsUMI to each end of a DNA duplex into the library preparation itself, avoiding the need for separate complex synthesis of dsUMI-carrying forked adapters (16). To achieve this, we developed OPUSeq, which has two main features. In this approach, a single-stranded UMI in the adapter is made double-stranded during the first steps of the PCR. At the same time, we maintain asymmetry of DNA ends necessary for Illumina library preparation by splitting the PCR into two stages.

The molecular details of the OPUSeq approach are shown in Figure 1A. Each adapter consists of two oligos that are easily synthesized by any commercial provider and simply annealed before ligation. The longer oligo contains a single-stranded six-base UMI and a binding region for Illumina primers (blue), while the shorter oligo consists of a sequence necessary for annealing. After ligation, two random versions of the adapter (exemplified here as ‘UMI A’ and ‘UMI B’) will be attached to the ends of each double-stranded DNA fragment. Ligated DNA is added to PCR mix 1, lacking dTTP and primer 2. During the first elongation step, the polymerase copies the UMI to the other strand but stops at the first encountered dA due to the lack of dTTP. This incomplete extension maintains asymmetric ends compatible with library PCR without using forked adapters. Next, DNA is denatured and primer 1 is annealed. Since sequences at each end of a strand are reverse complementary at this point, hairpin formation is possible, which would inhibit further amplification and diminish PCR efficiency. We have made two modifications to prevent this. First, we diversified the adapter annealing region by using a pool of four adapters with different annealing sequences (yellow–green in Figure 1A). Second, we added a blocking oligo with a *T*_m_ high enough to outcompete hairpin formation. After the first stage, the reaction is cooled to room temperature and mix 2 (containing the two missing PCR components) is added. As the reaction continues, primer 1 is fully extended and displaces the blocking oligo. This generates an asymmetric DNA molecule that is amplified by the following standard PCR cycles. Thus, despite seeming complexity, OPUSeq differs from a standard PCR protocol only by the inclusion of a short (2–3 min) pre-incubation and subsequent addition of the two missing PCR components.

OPUSeq is designed as a module that can be easily integrated into existing PCR-based Illumina library preparation workflows. We have successfully used it together with fragmentase- and sonication-based kits (KAPA HyperPlus and HyperPrep) with minimal modifications (Figure 1B). To demonstrate the ability of OPUSeq to generate high-quality sequencing libraries, we have applied it to human gDNA with input amount ranging from 5 ng to 1 μg. Since target enrichment is usually necessary when looking for low-frequency variants, we also tested OPUSeq performance with hybridization target capture using a few different panels up to 100 kb in size. Even using small DNA inputs, the capture efficiency was as good as that of the established protocol, with good coverage uniformity across a 56-kb panel covering a set of clinically relevant genes implicated in cancer (Figure 1C and 1D).

### Design of validation strategy for low variant fraction detection

**Figure 2.**
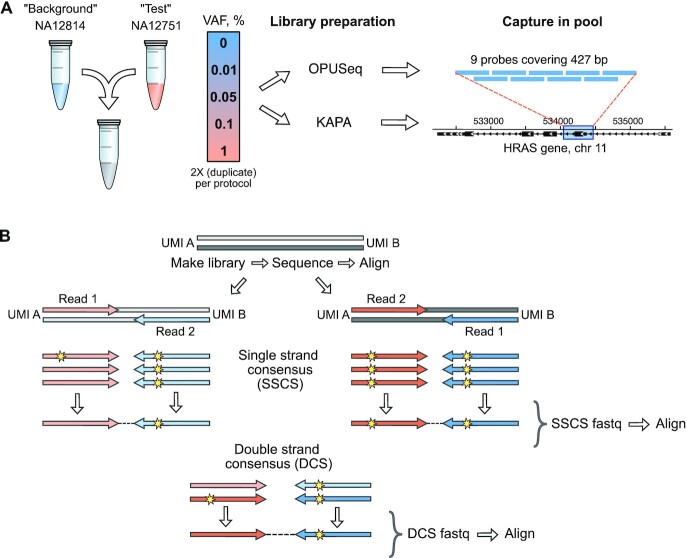
Experimental design and computational workflow for low VAF detection. (**A**) Validation experiment to demonstrate low VAF detection with OPUSeq. Two genotypes were assigned as ‘background’ and ‘test’. They were mixed at 0–1% VAF of ‘test’ in ‘background’ in duplicate. Each gDNA mix was split into two halves: one half was subjected to OPUSeq and the other to standard KAPA protocol. The resulting libraries were captured on probes covering 427 bp of the HRAS gene on chromosome 11. (**B**) Overview of the computational workflow for OPUSeq data analysis. After library PCR, each original strand of a DNA duplex gives rise to its own PCR product, and each of these results in two read families (reads 1 and 2). After aligning reads to the human genome, tag families are formed by grouping reads with the same genomic coordinates, orientation and UMI. The reads within each tag family are compared to form SSCS. Only bases that are present in over two-thirds of reads are kept, which excludes errors. Finally, SSCS from opposing strands are compared to form DCS.

In clinical applications such as MRD detection, the objective is to identify variants unique to the tumor that may be present at low fractions. To validate the applicability of OPUSeq to such situations, we tested it using DNA samples where the true variant fraction was known. We mixed defined amounts of DNA with known genotypes derived from the International HapMap Project. We assigned one genotype as ‘background’ (NA12814) and another as ‘test’ (NA12751) and assessed our ability to detect the SNPs of the test genotype when mixed into the background genotype at a series of VAFs from 0.01% to 1% (Figure 2A). In order to detect such low VAFs, at least 10 000× coverage is needed. One nanogram of human gDNA contains around 170 copies of each allele; therefore, in theory, 60 ng or more gDNA is required for 10 000 copies. However, not all molecules present in the sample are successfully transformed into a sequencing library—some are lost during library preparation. This can lead to poor efficiency in consensus making (see later). Therefore, in practice, a much larger DNA amount is necessary to ensure >10 000× unique coverage. We decided to use 1 μg DNA—the highest possible input in the KAPA HyperPlus Kit. Further, to sequence each strand many times (as is necessary for error correction) given such high input, a very high raw read coverage is needed. To achieve that, we focused on a very small target region—one exon of the HRAS gene, commonly mutated in cancer (24). In this region, our test genotype has two heterozygous SNPs that are absent in the background genotype (chr11:534197 C>T and chr11:534242 A>G). To enrich such a small region (427 bp), we used two rounds of hybridization capture, as previously performed for targeted Duplex-seq of ABL1 (25). We prepared gDNA mixes at each VAF in duplicate and made libraries using OPUSeq with a fragmentase-based protocol (KAPA HyperPlus). In parallel, we also prepared libraries from the same samples following the standard KAPA workflow.

For analysis of OPUSeq data, we established a computational workflow that utilizes both the dsUMIs and the start and end coordinates (genomic boundaries) to form DCS. We corrected UMIs using the grouping algorithm of UMI-tools (26) to ensure that even reads with errors in their UMI would be retained in the following analysis. We then formed consensus reads using a custom algorithm (see the ‘Materials and Methods’ section). Reads were grouped into tag families based on the start and end positions, UMI and read orientation. Each unique DNA fragment gives rise to four tag families: reads 1 and 2 from the each of the original (Watson and Crick) strands (Figure 2B). We compared the reads within each family to construct a final SSCS read. The four resulting SSCS reads are combined to make up the two reads of the DCS. Finally, we re-mapped the SSCS and DCS reads to the genome. To find single-nucleotide variants, we counted reference and variant base calls at each covered genomic position (we did not consider insertions or deletions). This computational workflow removes any errors and strand-specific DNA damage in the sequencing data while preserving true variants.

### OPUSeq duplex analysis reveals unexpected variants present at low VAF

**Figure 3.**
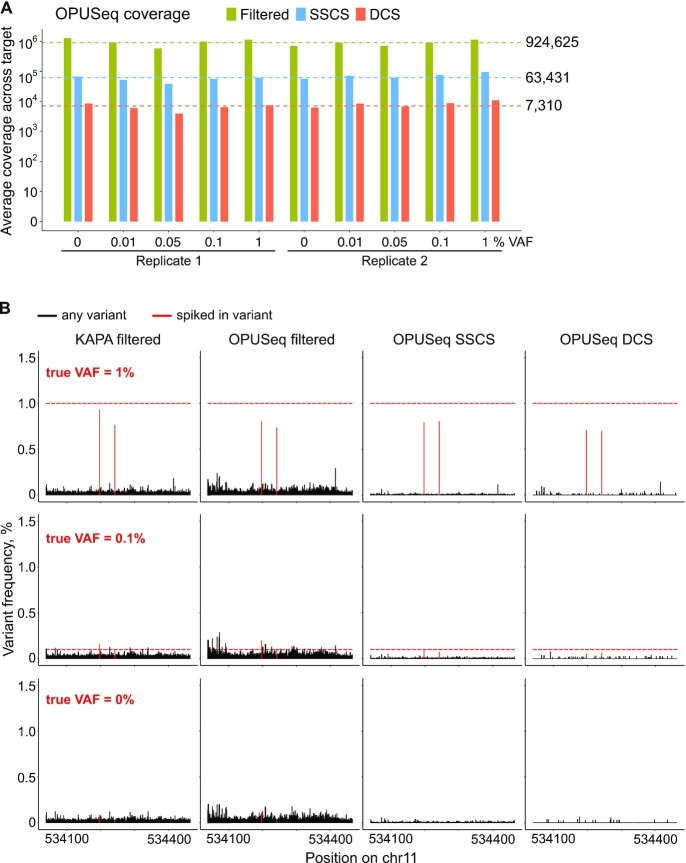
OPUSeq coupled with a fragmentase-based approach helps to remove most, but not all, spurious variants. (**A**) Log_10_-transformed average coverage across target by filtered, SSCS and DCS aligned reads in the 10 sequenced OPUSeq samples obtained using the fragmentase-based protocol. The dashed lines and numbers mark the cross-sample averages. (**B**) Variant frequencies (%) of all detected variants per position in the target region in KAPA filtered, OPUSeq filtered, OPUSeq SSCS and DCS reads (fragmentase protocol). The spiked-in variants are labeled in red. The red dashed lines mark the expected variant frequency of the spiked-in variants. The samples shown are from replicate 2.

We mapped both OPUSeq and standard KAPA reads to the human genome. The on-target rate was excellent, with OPUSeq performing slightly better than KAPA (Supplementary Figure S2A). We further processed OPUSeq reads with our full computational workflow to assess removal of errors and detection of the spiked-in variants. The final data output consisted of base counts from OPUSeq and KAPA mapped reads filtered by base and mapping quality (‘filtered’) as well as OPUSeq SSCS and DCS mapped reads. The average on-target coverage obtained at single-strand consensus level across OPUSeq samples was around 64 000. However, at duplex consensus level, it was only 7400, which limits the maximum sensitivity (Figure 3A). If one of the strands of an original DNA duplex is absent from the data, the DCS cannot be constructed, leading to a drop in coverage. One reason for the absence of some strands could be insufficient sequencing depth, but plotting single-strand family sizes (Supplementary Figure S2B) showed that sequencing deeper would be unlikely to recover more families. However, it is known that during library preparation, some strands completely drop out, and poor duplex recovery rates (percentage of SSCS converted into a DCS) have been observed with other approaches (19,23). Indeed, in our dataset, DCS recovery rate was around 22%.

The advantage of consensus over filtered mapped reads for calling low-frequency variants was obvious (Figure 3B). In filtered reads obtained with either OPUSeq or KAPA, all possible substitutions were detected at every position in the target region at VAFs ranging from 0.0003% to 0.26%. While these errors do not interfere with calling of 1% true VAF, variants displaying VAF below 0.5% would be difficult to call reliably. Many variant callers use *P*-values calculated by comparing the counts of reference and variant bases between paired tumor and healthy tissue samples to help in variant calling (27,28). We applied this kind of analysis to our data to check to what extent it would help separate true variants from the noise. However, due to the very high coverage, both true and spurious variants received extremely low *P*-values, making it impossible to confidently call even the 1% true VAF without false positives (Supplementary Figure S3). On the other hand, constructing single-strand consensus (corresponding to the use of a single-stranded UMI) removed the majority of these errors. Duplex consensus analysis further reduced the number of non-spiked-in detected variants to ∼40 per sample. Although these unexpected variants were detected at very low VAF (up to 0.14%), they could still obscure true variants of 0.1% and below. We were surprised by this result, since duplex consensus analysis should remove any variants not present in both strands of the original DNA fragment. However, a certain number of somatic mutations are naturally present in DNA, and the higher the unique coverage, the more of these will be detected. Therefore, we evaluated the number and frequency of identified variants in respect to coverage. We calculated variant incidence by dividing the number of variant bases by the total number of bases covered, excluding the positions with true variants. Variant incidence at duplex consensus level was around 2.6 × 10^–5^ (Supplementary Figure S4), which is two orders of magnitude higher than the estimated human somatic mutation rate of 3 × 10^–7^ (29).

### Use of fragmentase introduces spurious double-stranded variants in gDNA

Since the number of detected double-stranded variants was much higher than the somatic mutation rate, we decided to investigate their origin. To this end, we formulated two main hypotheses: that the DNA material actually contains a higher number of mutations or that mutations were introduced during the library preparation process. We used DNA derived from the HapMap project, which is extracted from Epstein–Barr virus-immortalized B lymphocytes (30) and propagated in culture for an unknown number of passages. Thus, one could speculate that these cells may have a higher mutation rate than healthy human tissue. Indeed, the substitution profiles of DCS variants (Supplementary Figure S5A and B) were similar to those reported for somatic mutations in the human body (31), with a prevalence of T>C and C>T transitions. Alternatively, during library preparation, the enzymatic fragmentation and/or ER process may introduce errors. We checked whether most variants were found near the ends of reads, as this could indicate an ER issue, but this was not the case (Supplementary Figure S5C). Gregory *et al.* recently discovered that the fragmentase enzyme mix induces single-nucleotide variants in up to 2% of reads (32). These spurious variants appear in reads that have a soft-clipped portion and are likely found in only one strand. However, we found no association between soft-clipping and DCS variants in our data. Additionally, errors present in one strand only will always be eliminated after duplex consensus analysis. We hypothesized that other errors might arise during the fragmentase treatment and the following ER, which might only be discovered by duplex consensus analysis.

**Figure 4.**
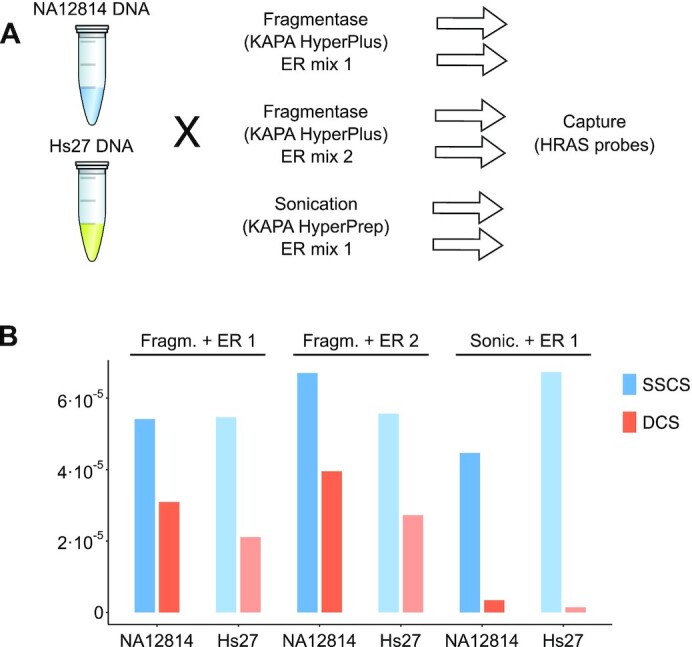
Spurious variants are much more abundant in samples treated with fragmentase. (**A**) Design of the experiment testing different fragmentation and ER methods as well as DNA input source. Libraries were made from NA12814 and Hs27 DNA using either KAPA HyperPlus with ER mix 1 or 2 or KAPA HyperPrep with ER mix 1 (see the ‘Materials and Methods’ section). They were captured on the same probes as in Figure 2 and sequenced. (**B**) The variant incidence (excluding the expected variants) for the six resulting samples at single-strand and duplex consensus levels.

We designed an experiment to test these two potential sources of error. We isolated gDNA from human neonatal fibroblasts (Hs27) and compared it to HapMap NA12814 DNA. Both DNA inputs were subjected to OPUSeq coupled with either the enzymatic fragmentation-based protocol (KAPA HyperPlus) using two alternative ER mixes (ER mix 1 or ER mix 2) or the sonication-based protocol (KAPA HyperPrep) (Figure 4A). As before, we performed capture on our HRAS probes. We sequenced the resulting libraries and constructed SSCS and DCS in the same way as mentioned earlier. Next, we computed variant incidence for each sample (Figure 4B). While SSCS variant incidence was similar across samples, DCS incidence clearly showed that fragmentase was responsible for a vast majority (90%) of double-strand errors found in a HyperPlus-generated dataset. Further, samples treated with the newer, fragmentase-optimized ER mix (ER mix 2) had slightly higher variant incidence compared to ER mix 1. Finally, NA12814 DNA did seem to have a somewhat higher mutational load than Hs27, but this difference was minor compared to the effect of fragmentase versus sonication. Thus, our results indicate that fragmentase treatment induces a substantial number of difficult-to-remove double-strand artifacts in DNA sequencing data, albeit at a very low VAF.

### Sonication-based OPUSeq enables the accurate identification of low VAFs

Having identified that the use of fragmentase introduces spurious variants at double-strand level, we evaluated the performance of OPUSeq in variant calling using the same experimental design (Figure 2A), but now with the sonication-based protocol. In this second experiment we also decided to decrease the starting amount of DNA, as we suspected that the 1 μg input we used previously could overload the library preparation or capture and lead to poor library conversion. Indeed, when the input material was reduced to 200 ng, the library conversion efficiency was much improved: unique (DCS) coverage was only 1.7-fold lower despite a 5-fold smaller input (Supplementary Figure S6A). This was also demonstrated by the increase in DCS recovery from 22% to 43%.

**Figure 5.**
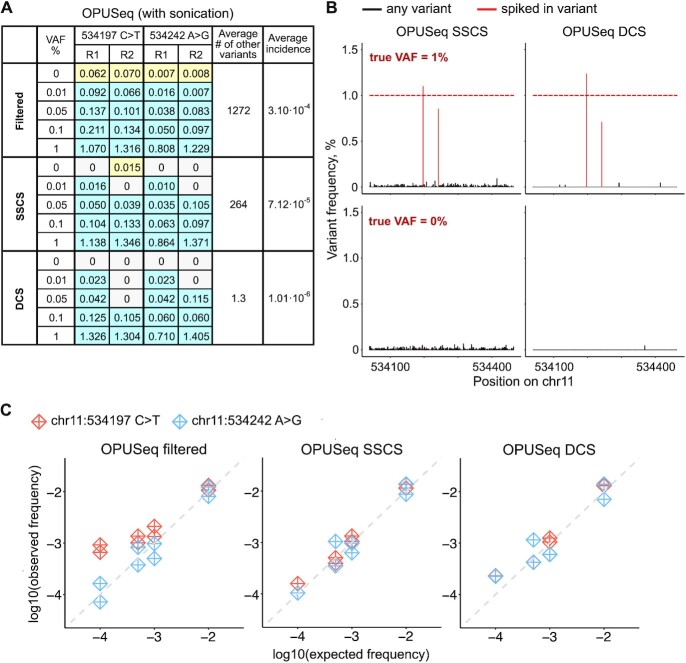
OPUSeq coupled with a sonication-based protocol shows better performance in error removal. (**A**) Summary table with results obtained from OPUSeq with sonication. Each colored-in cell shows the observed VAF (in %) of the specified spiked-in variant in each sample (for expected VAF of 0–1% and replicates 1 and 2). Blue: variants that were expected and detected. Yellow: variants that were erroneously detected in 0% VAF samples, where they should not be present. For each method of analysis (filtered reads, SSCS and DCS), the table shows the average number across all 10 samples of all unexpected (‘other’) detected variants. The last column shows the average unexpected variant incidence per method. (**B**) Plots as in Figure 3B, but for OPUSeq with sonication (samples shown are from replicate 1). (**C**) Log_10_-transformed observed versus expected VAFs of the spiked-in variants.

As predicted, the number of variants detected at the double-strand level was greatly reduced when using sonication instead of fragmentase. However, we observed an increase in variant incidence within 7 bp of the ends of reads (Supplementary Figure S6B), which indicated the presence of sonication or ER artifacts. Therefore, we trimmed 7 bp from each end before calling final variants. We summarized the observed frequency of spiked-in variants and the across-sample averages of the number of unexpected variants and variant incidence in Figure 5A. Visual representations of the variant frequencies per position and the relationship between observed and expected VAFs are provided in Figure 5B and C. Even after forming double-strand consensus, all spiked-in VAFs were detected in at least one replicate. The incidence of unexpected DCS variants was now 1 × 10^–6^, which is not far from somatic mutation rate and could plausibly be the biological reality in this target region and cell line. Thus, we concluded that OPUSeq in combination with a sonication-based protocol is capable of effectively removing errors and correctly calling variants down to the lowest tested VAF of 0.01%.

## DISCUSSION

Accurate detection of genomic alterations is essential for cancer patient management and precision medicine. Due to factors such as intratumoral heterogeneity or treatment-induced mutations, many clinically actionable variants are present at low VAF (<1%) (1). However, sequencing errors and DNA damage limit our ability to accurately quantify low VAFs. Here, we presented a simplified approach enabling error correction by the generation of duplex consensus (OPUSeq). OPUSeq is a simple and straightforward library preparation protocol using readily available commercial adapters that can be easily integrated into existing PCR-based library preparation workflows. It introduces dsUMIs in the same reaction tube as the PCR and is compatible with standard target capture approaches. We demonstrate that OPUSeq can remove any sequencing errors or single-strand changes in the DNA, preserving the true double-stranded variants at least down to 0.01% VAF using 200 ng human gDNA. In addition, although we focused on detection of single base substitutions, we noted that there is a six-base heterozygous deletion in our HRAS target region in the ‘test’ genotype NA12751. We checked whether this deletion was successfully detected in our OPUSeq duplex consensus data. Indeed, it was observed in all 1% and 0.1% VAF samples (Supplementary Figure S7), albeit at a somewhat lower frequency than the single base substitutions. As our computational pipeline was not optimized for indel detection, we believe that this aspect can be improved in the future, allowing OPUSeq to be applied to structural variation detection.

Since we developed OPUSeq with a potential future clinical application in mind, we tried to minimize any additional steps that could hinder this. Fragmentase-based kits are commonly used in clinical sequencing workflows, since this obviates the necessity of expensive sonicator instruments and decreases required steps. Thus, we first validated our method using an enzymatic fragmentation-based kit. Surprisingly, after forming DCS, we discovered an unexpected number of double-stranded variants in the data (2.6 in 10^5^ bases were mutated) that prevented the accurate identification of low VAFs. These variants cannot be easily detected with standard approaches, but became apparent with our duplex consensus analysis. Interestingly, those errors were not observed when a sonication-based approach was used instead of a fragmentase-based one. Recently, it has been reported that the use of enzymatic fragmentation cocktails can induce the formation of library molecules containing regions of nearby DNA from opposite strands in up to 2% of the molecules (32). In those cases, the introduced errors are associated with inversions at the read boundaries and can be removed computationally. In contrast, the errors that we report here are present at lower frequencies, interfering with calling of variants at fractions of 0.1%. Since they occur along the whole read length and are present in both strands of the original DNA molecule [unlike the errors observed in (32)], they cannot be computationally removed.

It should be noted that we have only tested one fragmentase formulation, and different enzyme mixes from other manufacturers may differ in regard to the observed artifacts. The composition of KAPA fragmentase is proprietary, and a precise mechanism is hard to define. However, we speculate that it is likely to contain a nickase and a nuclease that cleaves DNA at nicks, as is the case for other manufacturers. We hypothesize that, after treatment, some DNA nicks might remain across the entire length of the DNA fragment. During ER, the 3′ ends at the nicks may get extended and replace the remaining original strand (as in nick translation). If DNA damage is present in the other strand, a substitution may be introduced, generating a double-stranded variant.

To avoid such fragmentase-induced variants, we showed that sonication can be used instead. It has long been known that sonication induces changes to DNA via several mechanisms (33,34). However, we observe the rate of spurious double strand after sonication to be at least 10-fold lower than after fragmentase treatment. In addition, a majority of such errors are expected to aggregate toward the ends of reads, which is what we observe in our dataset (Supplementary Figure S6B). If the ends are trimmed, most sonication damage can be removed. However, the use of sonication for clinical genomic applications is not problem-free. For example, it can lead to significant DNA losses during library preparation. This can make sonication unfeasible in those cases where only a limited amount of patient material is available. Thus, clinical laboratories should balance the respective advantages and limitations of the selected method depending on available material and intended application.

In summary, we have presented here a novel method for simplified detection of low VAF at least down to 0.01%. Using this method, we have revealed that the use of fragmentase-based strategies introduces double-stranded artifacts in gDNA libraries interfering with the identification of VAF around 0.1% and below. This result should be taken into consideration when designing protocols for low-frequency variant calling.

## DATA AVAILABILITY

All code used in this study can be found at https://github.com/PelechanoLab/OPUSeq_tools.

The data for this study have been deposited in the European Nucleotide Archive (ENA) at EMBL-EBI under accession number PRJEB51234 (https://www.ebi.ac.uk/ena/browser/view/PRJEB51234).

## Supplementary Material

lqac048_Supplemental_File

## Notes

Present address: Jingwen Wang, BenevolentAI Ltd, 4–8 Maple Street, London W1T 5HD, UK.
