# Supplementary material for: OPUSeq simplifies detection of low-frequency DNA variants and uncovers fragmentase-associated artifacts
Source: NAR Genom Bioinform. 2022 Jun 27;4(2):lqac048. doi: 10.1093/nargab/lqac048 (PMC9235115; doi:10.1093/nargab/lqac048)
Supplement: lqac048_Supplemental_File [file lqac048_supplemental_file.pdf]

SUPPLEMENTARY MATERIAL

**Name**	**Sequence**	**Purification**
Adapter_Fw_1	GACTGGAGTTCAGACGTGAGCTCTTCCGTTCTBBBBBBGCCAATGC*T	HPLC
Adapter_Fw_2	GACTGGAGTTCAGACGTGAGCTCTTCCGTTCTBBBBBBTCACTGCG*T	HPLC
Adapter_Fw_3	GACTGGAGTTCAGACGTGAGCTCTTCCGTTCTBBBBBBTTGTGCGT*T	HPLC
Adapter_Fw_4	GACTGGAGTTCAGACGTGAGCTCTTCCGTTCTBBBBBBAGCACATT*T	HPLC
Adapter_Rv_1	Phos-GCATTGGC	DST
Adapter_Rv_2	Phos-CGCAGTGA	DST
Adapter_Rv_3	Phos-ACGCACAA	DST
Adapter_Rv_4	Phos-AATGTGCT	DST
PE1_ MM_MPX_[index#] (PCR primer 1)	AATGATACGGCGACCACCGAGATCTACAC**[i5]**ACACTCTTTCCCTACACGACGCTCTTCCGTTC*T	HPLC
PE2_MPX_[index#] (PCR primer 2)	CAAGCAGAAGACGGCATACGAGAT**[i7]**GTGACTGGAGTTCAGACGTGTGCTCTTCCGATC*T	HPLC
R1_seq_MM	ACACTCTTTCCCTACACGACGCTCTTCCGTTC*T	HPLC
Block	CGGAAGAGCTCACGTCTGAACTC-Inv(dT)	DST

Supplementary Table 1. Sequences of all the oligonucleotides used for OPUSeq library preparation and sequencing. The bases which have been modified compared to the original Illumina adapter or primer design are marked in red. Phos = 5’ phosphorylation; Inv(dT) = a 3’ inverted dT base which prevents elongation and degradation; star stands for a phosphorothioate bond; [i5] and [i7] denote the location of multiplexing indexes; DST = desalting; HPLC = high performance liquid chromatography.

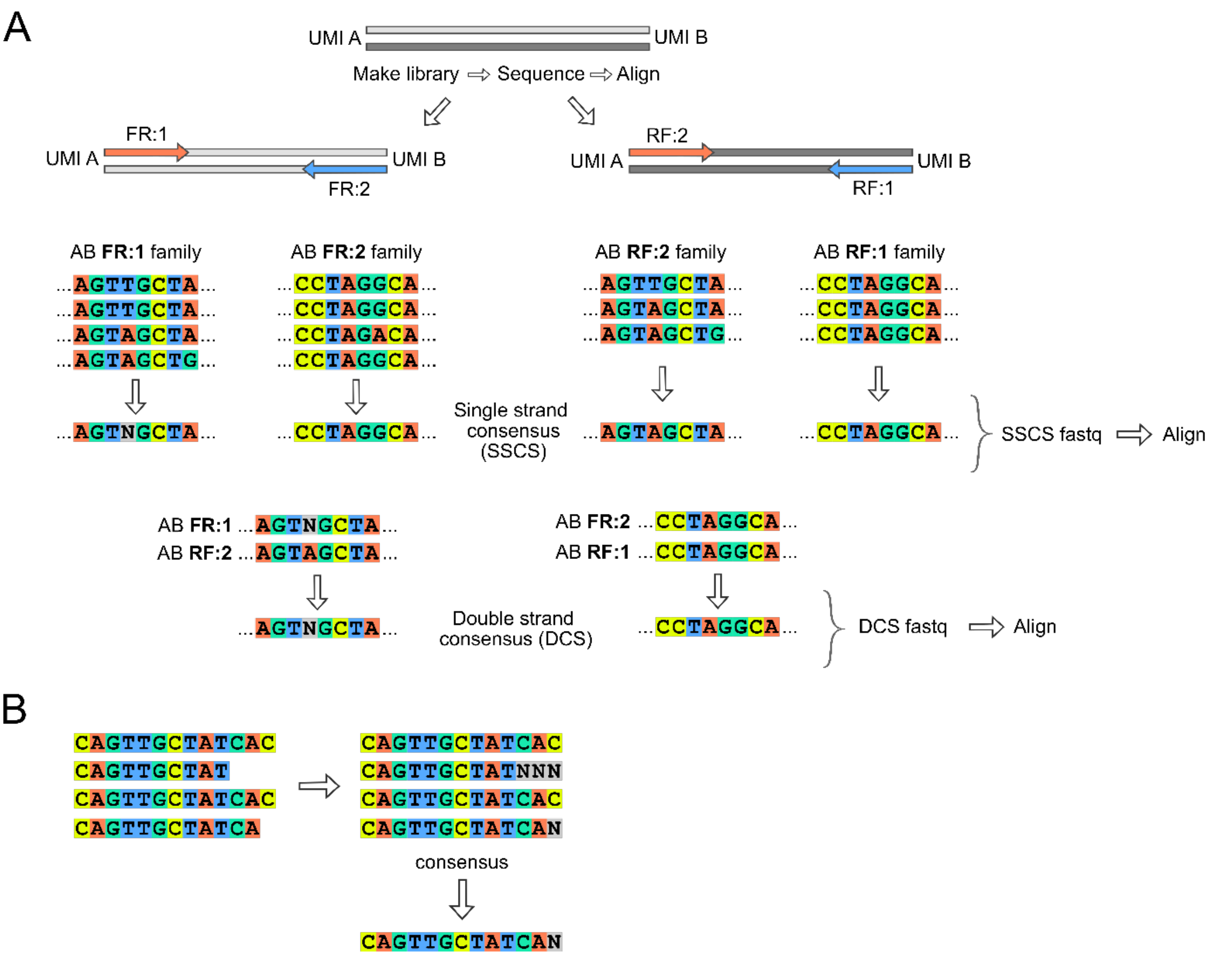

Supplementary Figure 1. Detailed computational workflow for consensus making. (**A**) An overview of the consensus making algorithm. After library PCR, each original strand of a DNA duplex gives rise to a PCR product with different orientation, and each of these results in two read families (read 1 and 2). Reads from each of the original strands are distinguished by their orientation. Watson strand reads will be oriented as "read 1 forward, read 2 reverse" (FR) and Crick strand reads will have the opposite orientation (RF). After initial alignment and UMI correction by UMI tools, we label reads with the corrected UMIs from both ends, the genomic coordinates, and the orientation. We group reads into tag families based on these three factors and the read number. We retain only families with at least three members. Thus, four tag families can maximally be obtained from each original DNA duplex. We then compare reads from each family base by base to form consensus. If over 2/3 of reads have the same base call, this base is written to consensus sequence; if not, N is written. The resulting single-strand consensus sequences (SSCS) are then paired up with the SSCS from the opposite strand (FR:1 with RF:2, FR:2 with RF:1) and consensus is formed between these to obtain duplex consensus sequences (DCS). Since only two bases are compared at each position, a base call is made only if both match. Finally, the resulting SSCS and DCS are written to FASTQ files and re-mapped to the genome using local alignment. (**B**) Schematic explaining how we deal with reads of different lengths within the same tag family. N is added to the ends of shorter reads until all reads reach the same length. This eliminates the possibility of base calls at the end of consensus sequences which are only supported by one or two reads. It also ensures that all consensus base calls within the same tag family are made based on the same number of input bases.

A

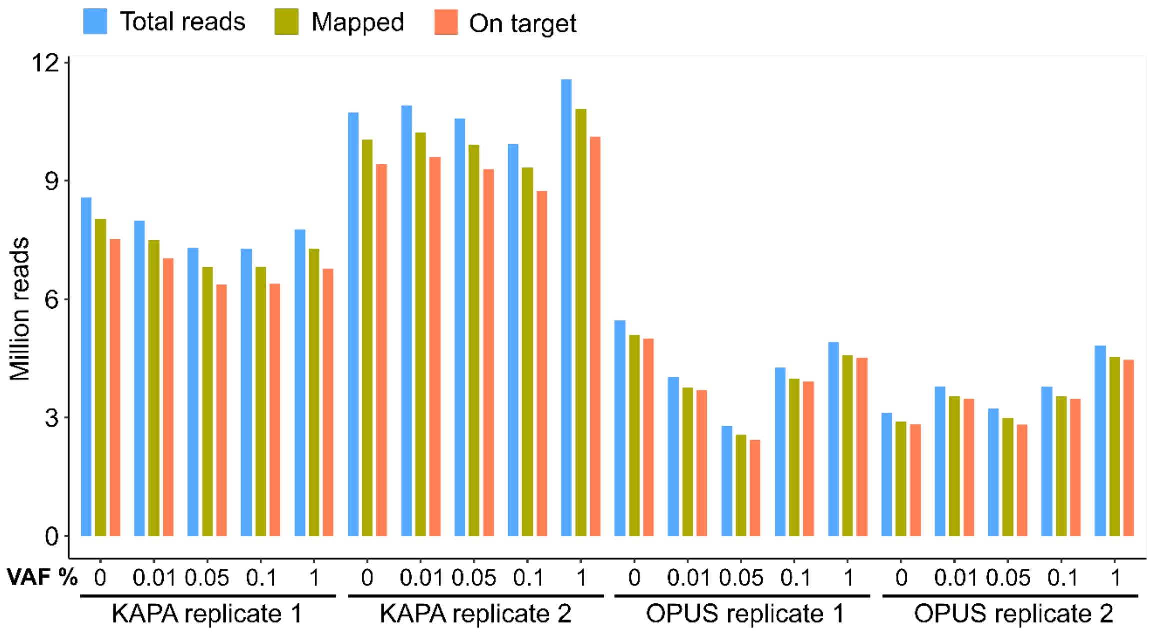

B

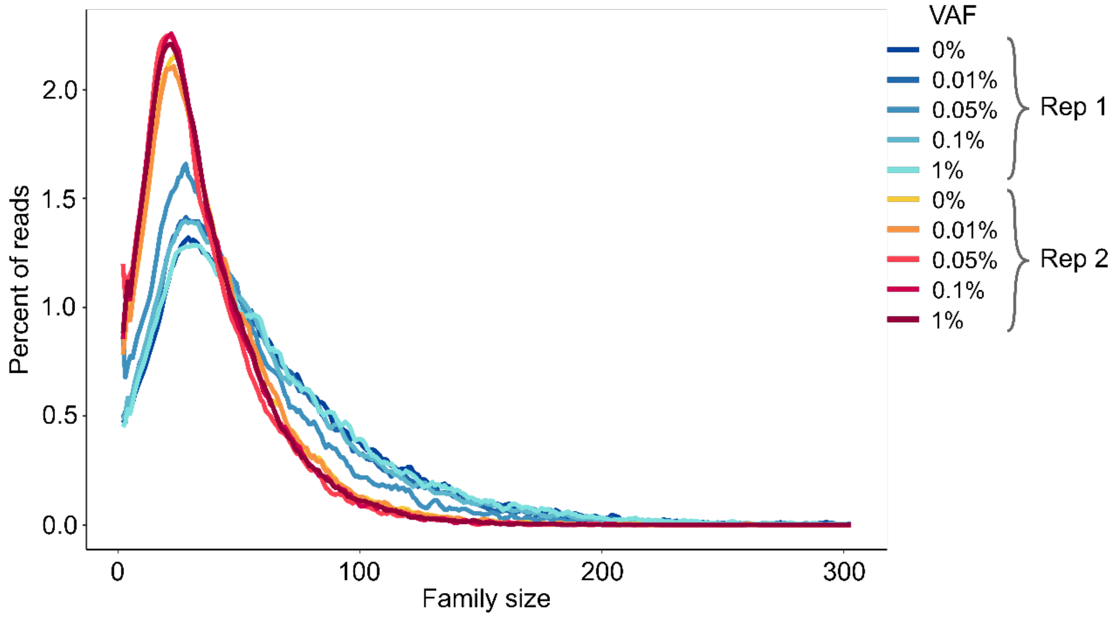

Supplementary Figure 2. Mapping statistics and family size metrics for the fragmentase-based approach. **(A)** The number of total, mapped, and on target reads in each sample obtained with KAPA or OPUSeq using the fragmentase-based protocol. **(B)** Percent of reads in families of different sizes in the OPUSeq dataset using the fragmentase-based protocol. The datapoint for family size of one read was omitted to be able to plot the smoothened line. (The fraction of reads in such families was between 0.4 to 3.8% depending on sample.) The smoothened line was then plotted using the rolling mean with a window width of 4. The percentage of reads in families of size 1 or 2 (below minimum family members threshold) is between 2.5% and 7.3%, depending on sample.

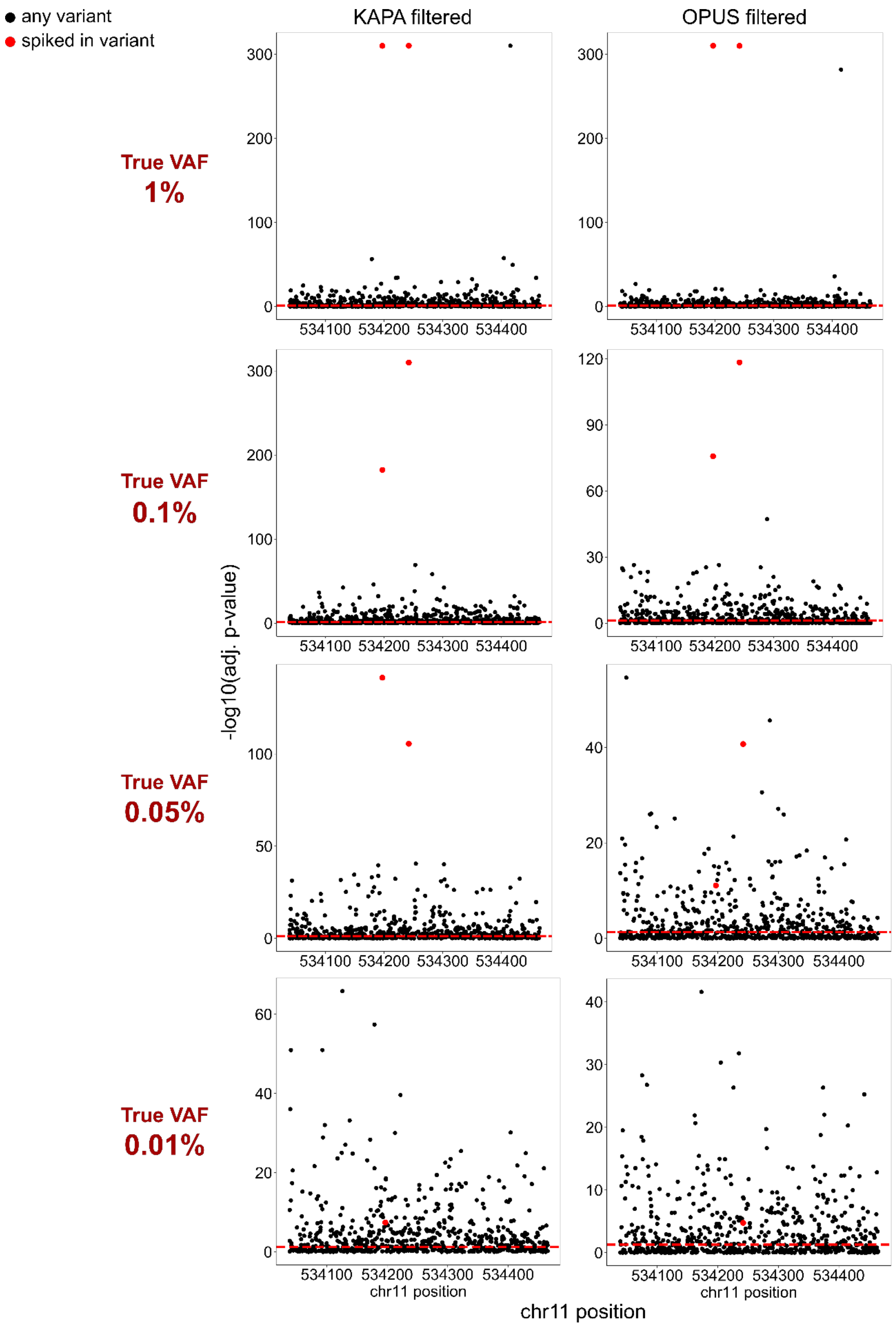

Supplementary Figure 3. Adjusted Chi-squared values for each detected variant in the filtered KAPA and OPUS reads (replicate 2). The samples with 0% VAF were set as "background" and the rest as

“test”. Each “test” sample was compared to the “background” from the same replicate. Only variants with a higher frequency in the “test” than “background” sample were considered. For each such variant, counts supporting reference and variant bases were used to perform a Chi-squared test. The resulting p-values were corrected using the Benjamini-Hochberg method. Finally, -log10(adjusted p-values) were plotted against genomic position. The red dashed lines denote the threshold of 0.05 adjusted p-value (5% false discovery rate). Around 300 variants in each sample passed this threshold. Although the p-values for the spiked-in variants were smaller than the remaining variants in many cases, we do not see a way to formally define a significance threshold so that it would separate only these “true” variants from the rest. This is a result of the extremely high coverage, since large input numbers to the Chi-square test lead to very small p-values.

A

OPUS-seq (with fragmentase)

	VAF %	534197 C>T		534242 A>G		Average # of other variants	Average incidence
		R1	R2	R1	R2		
Filtered	0	0.068	0.075	0.008	0.007	1272	$5.80 \cdot 10^{-4}$
	0.01	0.081	0.074	0.019	0.015		
	0.05	0.117	0.107	0.045	0.042		
	0.1	0.097	0.168	0.072	0.089		
	1	0.757	0.787	0.734	0.723		
SSCS	0	0.004	0.005	0	0	495	$6.61 \cdot 10^{-5}$
	0.01	0.007	0.010	0.004	0.011		
	0.05	0.046	0.039	0.038	0.036		
	0.1	0.034	0.091	0.060	0.072		
	1	0.799	0.782	0.812	0.807		
DCS	0	0	0	0	0	39.8	$2.56 \cdot 10^{-5}$
	0.01	0	0	0	0.022		
	0.05	0	0.035	0.044	0.052		
	0.1	0	0.056	0.028	0.061		
	1	0.829	0.708	0.759	0.695		

B

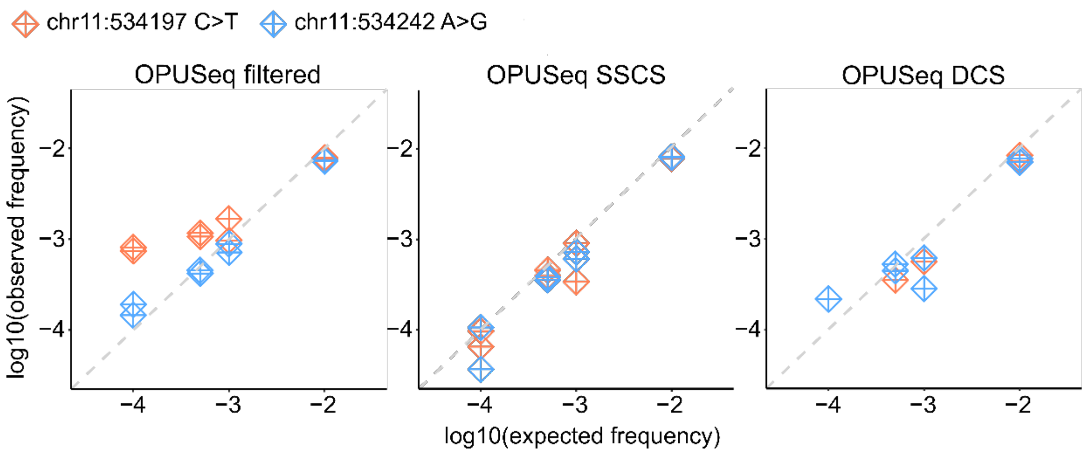

Supplementary Figure 4. Detailed results from OPUSeq with fragmentation. (**A**) Summary table for results obtained using OPUSeq with fragmentase-based protocol. Each colored-in cell shows the observed VAF (in %) of the specified spiked-in variant in each sample (for expected VAF of 0 to 1% and replicates 1 and 2). Blue: variants which were expected and detected. Yellow: variants which were erroneously detected in 0% VAF samples, where they should not be present. For each method of analysis (filtered reads, SSCS, DCS), the table shows the average number across all 10 samples of all unexpected ("other") detected variants. The last column shows the average unexpected variant incidence per method. (**B**) Log10-transformed observed vs. expected VAFs of the spiked-in variants.

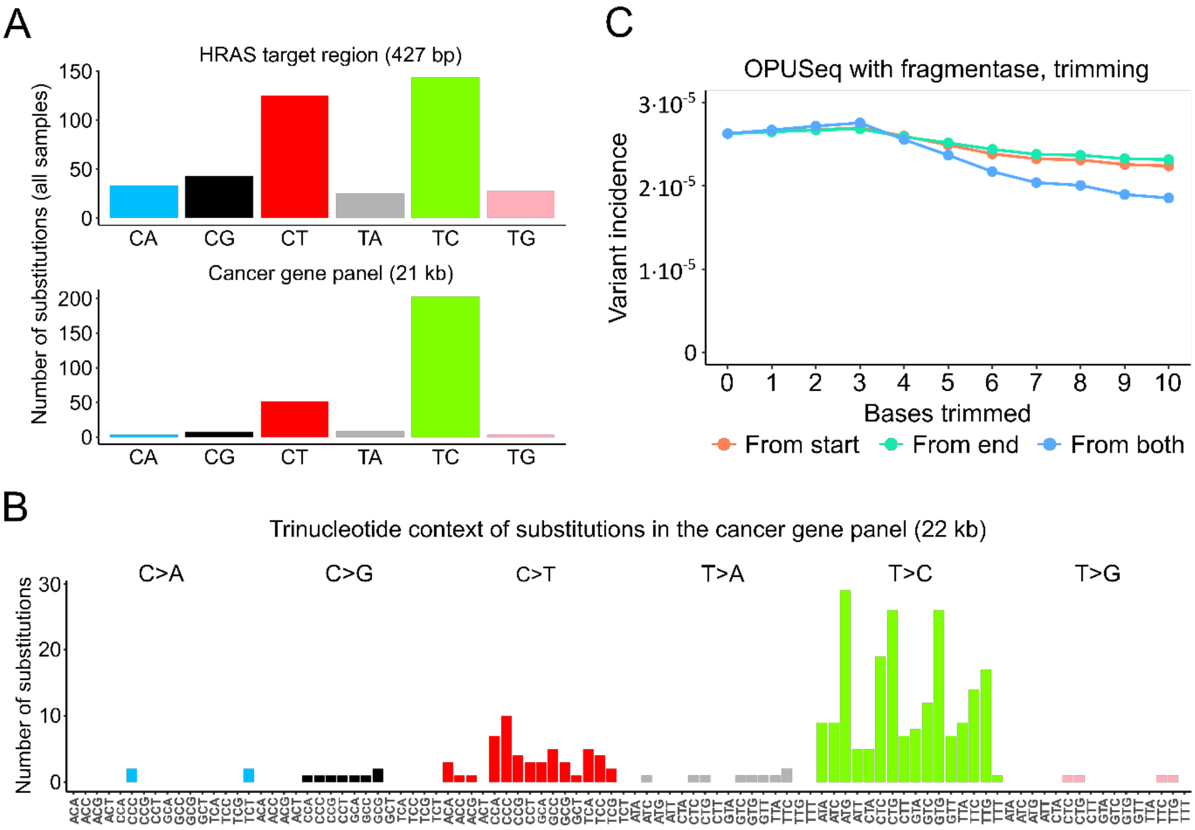

Supplementary Figure 5. Characterization of fragmentase-induced artifacts. (**A**) Substitution profiles in duplex consensus data obtained from OPUSeq with fragmentase. Top, the counts of substitutions (except germline and spiked-in variants) in all ten samples from the VAF series experiment captured on the HRAS target region (427 bp). Bottom, counts of non-germline substitutions in five samples which were captured on a larger cancer gene panel (22 Kb target region, same panel as in Figure 1). Since the libraries were prepared from 5 ng DNA for these samples, there were many fewer substitutions detected per position. However, given the much larger size of the capture panel, this mutational profile is more informative. (**B**) Substitutions broken down by trinucleotide context in the same five samples captured on the 21 kb panel. There seems to be a preference for G after T in T>C transitions. (**C**) Average variant incidence at duplex consensus level in the OPUSeq dataset (fragmentase-based protocol) calculated after trimming up to 10 bases from either start, end, or both sides of mapped reads using bamUtil.

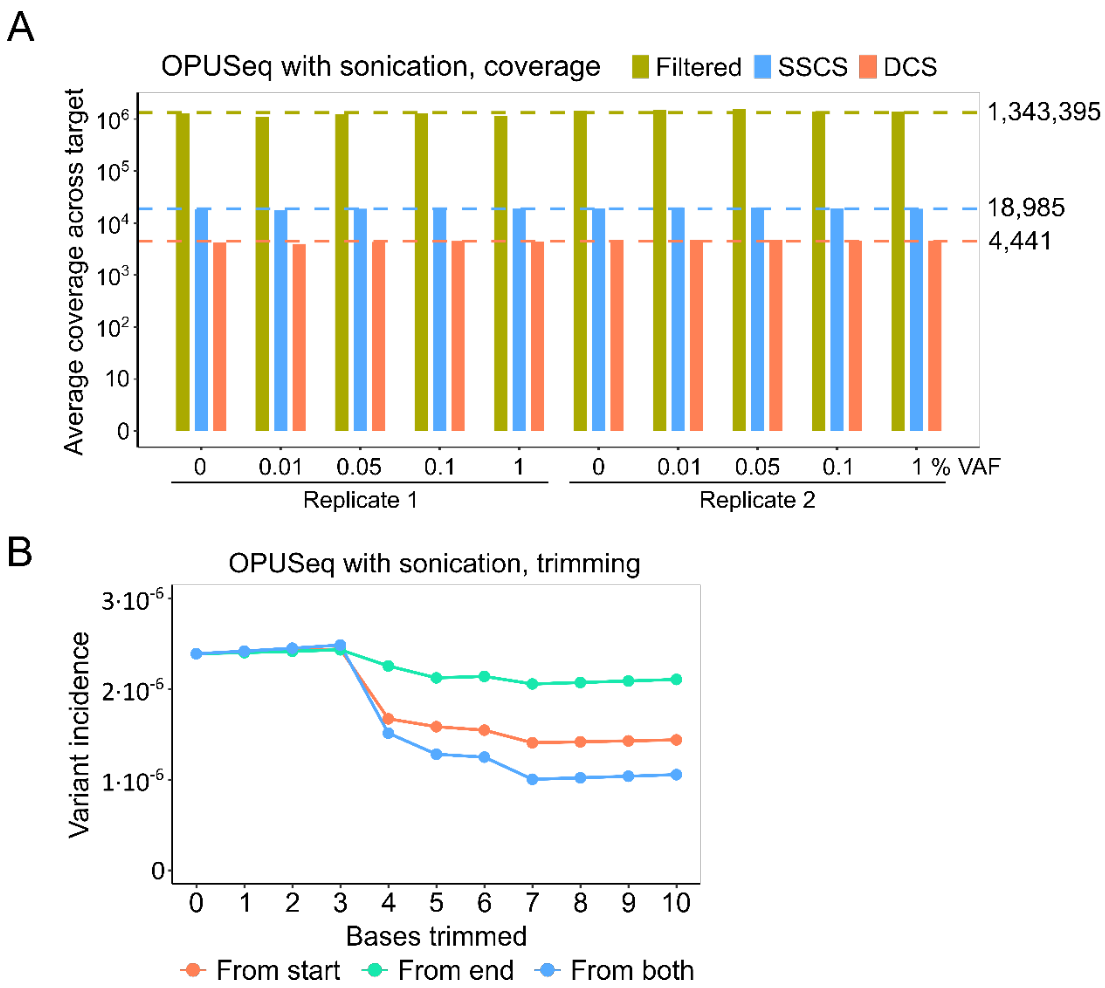

Supplementary Figure 6. Coverage for OPUSeq with sonication and the effect of read trimming on variant incidence. (**A**) log10-transformed average coverage across target for filtered, SSCS, and DCS aligned reads in the ten sequenced OPUSeq samples obtained using the sonication-based protocol. The dashed lines and numbers mark the cross-sample averages. (**B**) Average variant incidence at duplex consensus level in the OPUSeq dataset (sonication-based protocol) calculated after trimming up to 10 bases from either start, end, or both sides of mapped reads using bamUtil.

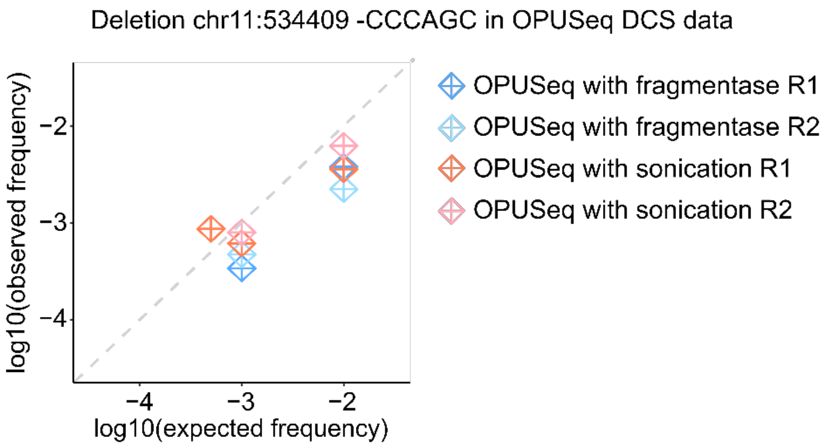

Supplementary Figure 7. Log-transformed observed vs. expected frequencies of a six-base deletion (heterozygous in the "test" genotype NA12751) in duplex consensus OPUSeq data from VAF dilution experiments.
